# Determining Contextual Factors for a Heart Failure Self-Care Intervention: A Consensus Delphi Study (ACHIEVE)

**DOI:** 10.1177/10901981211043116

**Published:** 2021-10-04

**Authors:** Amanda Whittal, Isabell Ehringfeld, Paula Steinhoff, Oliver Rudolf Herber

**Affiliations:** 1 Heinrich Heine University Düsseldorf, Düsseldorf, Germany; 2 Witten/Herdecke University, Witten, Germany

**Keywords:** behavior change, complex intervention design, consensus Delphi study, heart failure, key stakeholder, self-care

## Abstract

There is a rising recognition of the crucial role self-care plays in managing heart failure (HF). Yet patients often have difficulties implementing ongoing self-care recommendations into their daily lives. There is also recognition of the importance of theory for successful intervention design, and understanding of key factors for implementation so interventions fit a given context. Local key stakeholders can provide valuable insights to help understand relevant context-specific factors for intervention implementation. This study sought to engage stakeholders to explore and determine relevant contextual factors needed to design and facilitate successful implementation of an HF self-care intervention in the German health care system. A ranking-type Delphi approach was used to establish consensus from stakeholders (i.e., clinicians, patients, policymakers/potential funders) regarding eight factors (content, interventionist, target group, location, mode of delivery, intensity, duration, and format) to adequately define the components and implementation strategy of the intervention. Seventeen participants were invited to participate in the first Delphi round. A response rate of 94% (16/17) was achieved and maintained for all three Delphi rounds. Stakeholder consensus determined that nurses specializing in HF are the most appropriate interventionists, target groups should include patients *and* carers, and the intervention should occur in an outpatient HF clinic, be a mixture of group and individual training sessions, and last for 30 minutes. Sessions should take place more frequently in the beginning and less often over time. Local stakeholders can help determine contextual factors that must be taken into account for successful delivery of an intervention. This enables the intervention to be developed and applied based on these factors, to make it suitable for the target context and to enhance participation to achieve the desired outcomes.

Cardiovascular diseases (CVDs) are the leading cause of death worldwide (World Health Organization, 2017). Heart failure (HF), one of the conditions of CVD, is characterized by reduced cardiac output and/or increased intracardiac pressure resulting from structural and/or functional cardiac abnormality (Ponikowski et al., 2014). HF affects an estimated 38 million people globally (Braunwald, 2015) and is associated with increased hospitalization and mortality (Christ et al., 2016) as well as reduced quality of life (Chen et al., 2018). In Germany, HF represents a significant public health burden (Holstiege et al., 2018), with a national prevalence of 3.4% of people with statutory health insurance, a compulsory insurance in Germany covering about 90% (70 million) of the German population (Bundesgesundheitsministerium, 2021). There is a rising awareness of the need for specialized treatments for patients with HF and the crucial role self-care plays in managing the condition (Riegel & Moser, 2018).

Self-care refers to an individual’s ability to engage in symptom monitoring and management, treatment adherence, and lifestyle changes necessary to manage their illness, such as daily weighing, fluid restriction, or regular physical exercise (Riegel et al., 2017). Adhering to recommended self-care behaviors can substantially contribute to reducing illness symptoms, hospital admissions, and mortality, and improving quality of life for people living with HF (Riegel & Moser, 2018). Despite these positive effects, many patients fail to implement ongoing self-care behaviors in their daily lives (Chew et al., 2019). Worldwide, as many as 50% of patients fail to engage in regular self-care behaviors like daily weighing or adequate physical exercise (Jaarsma et al., 2013), suggesting a need for methods to improve HF self-care adherence.

Interventions to change health behaviors are complex (Kelly & Barker, 2016), and self-care-focused interventions aiming to improve outcomes for patients diagnosed with HF have shown inconsistent effectiveness (Jonkman et al., 2016). This may be partly because they were not theory based or well described for particular settings. A review of interventions promoting self-care of patients with HF found that few studies used explicit, theory-based interventions and the majority lacked specification of the mechanisms employed to improve self-care (Albano et al., 2014; Barnason et al., 2012). The result has been increased recognition of the importance of theory for successful intervention design (Prestwich et al., 2015).

Description standards for complex interventions need to be adhered to so that an intervention can be replicated. Davidson et al. (2003) outline eight factors for this purpose: (1) content of the intervention, (2) characteristics of the interventionist, (3) characteristics of the target population, (4) delivery location, (5) mode of delivery, (6) intensity, (7) duration, and (8) format. These factors are components of an intervention that should be determined for a particular context, since understanding the relationship between intervention and context is crucial (Craig et al., 2018). Intervention development and implementation should therefore involve key stakeholders to bring a contextual perspective (Eldredge et al., 2016). Even if an intervention is well described and has a strong theoretical basis, it will still fail to have a significant impact if local circumstances have been overlooked (O’Cathain et al., 2019).

With the help of key stakeholders, this study aims to define the eight factors described by Davidson et al. (2003) to successfully implement a theory-based HF self-care intervention in the German health care system (Herber et al., 2018). Expert interviews with three major stakeholder groups were conducted previously: (1) clinicians, (2) patients, and (3) policymakers and potential funders (Herber et al., 2021). Among other things, stakeholders were asked to elucidate their preferred choices for each of the eight factors needed for developing a full intervention by sharing their views on the most important contextual factors to facilitate the implementation of a theory-based HF intervention in Germany (Herber et al., 2021). In this study, the Delphi approach was used to reach consensus among these key stakeholders regarding the eight factors to adequately define the intervention for the German context.

## Method

### Study Design

A ranking-type Delphi study (Strasser, 2019) was selected to obtain consensus of opinions among key stakeholders (experts) concerning the eight factors for intervention implementation (Davidson et al., 2003). The Delphi method allows a group of experts to reach consensus in a way that gives equal weighting to each participant and maintains anonymity. Delphi studies have been widely used in health research to arrive at a final consensus (Jünger et al., 2017). For instance, they have been used successfully in previous studies to obtain expert consensus on intervention factors for reducing depression and anxiety (de Manincor et al., 2015), barriers and facilitators for interventions (Hanssen et al., 2021), and the feasibility of workplace health promotion interventions (Perry et al., 2017). The Delphi approach uses rounds of questions in which each round builds on the responses from the previous round. Consensus is reached when a predefined level of agreement is achieved. The Delphi encompassed three rounds and complied with the “Conducting and REporting of DElphi Studies” (CREDES) guidelines (Jünger et al., 2017). The study was approved by the ethics committee of the Medical Faculty of the Heinrich Heine University Düsseldorf, Germany (Ref #: 2018-30).

### Delphi Expert Panel

Eighteen key stakeholders who had previously participated in semistructured expert interviews about intervention implementation were invited to take part in the Delphi study (Herber et al., 2021). Key stakeholders were selected according to the following criteria: (1) expertise in HF, (2) diverse perspectives, (3) responsibility and authority to facilitate the implementation, (4) influence, and (5) commitment (Eldredge et al., 2016). All the stakeholders provided written informed consent. However, one stakeholder (a policymaker) participated in the expert interview but refused subsequent involvement in the Delphi questionnaire due to time constraints caused by COVID-19.

### Delphi Questionnaire Development

A questionnaire was developed in which stakeholders could select response items for Factor 1 and rank response options for Factors 2 to 8. The first-round Delphi questionnaire (Supplemental Appendix 1) comprised responses from stakeholders obtained during the interviews regarding the eight factors (Herber et al., 2021). For Factor 1 (content of the intervention), ambivalent behavior change techniques (BCTs) on which stakeholders expressed different opinions during the interviews were included to achieve a clearer vote. There were 11 BCTs in total: digital resources, teach-back method, involving a psychologist for additional support if necessary, self-help groups, differentiation of symptoms from other comorbidities, monitoring feelings, reattribution, teaching to read food labels, Post-It reminders on the fridge, motivational interviewing, and cognitive behavioral therapy. Hence, the Round 1 questionnaire comprised yes/no responses to either include or exclude certain BCTs. A comprehensive list depicting all BCTs to enhance self-care in people with HF can be found here (Whittal et al., 2020). For Factors 2 to 8, all the responses proposed during the interviews were included in the Delphi questionnaire and required ranking according to the stakeholders’ preference. To help stakeholders consider their decisions, response options were juxtaposed with evidence demonstrating the impact of different factors on self-care outcomes for individuals with HF or CVD from high-quality quantitative Cochrane reviews and/or qualitative metasyntheses for all eight factors (Moher et al., 2009). Where available, the scientific evidence was presented alongside the interview responses in a visual matrix following the example of Lovell et al. (2008). To minimize bias, the order of response items for each factor was randomized for each participant (Jünger et al., 2017; Strasser, 2019) using an online computer software (https://onlinerandomtools.com/shuffle-lines). The questionnaire was designed with Adobe Acrobat “Prepare Form,” to create a fillable PDF document that participants could open, complete, and return via email.

### Delphi Questionnaire Piloting

The questionnaire was piloted by two experienced researchers (B.R., S.W.) to ensure its user-friendliness. In addition, two nurse practitioners (A.H., C.D.) went through the questionnaire using the think-aloud method—verbalizing their thoughts as they completed it (Eccles & Arsal, 2017). Feedback from the piloting provided confirmation that the instructions for participants were very clear. Recommendations included underlining some words for emphasis, reducing the amount of text, and changing the order of some questions to avoid biasing participants to think in a certain way. All of these suggested changes were incorporated.

### Data Collection

The three rounds of data collection took place between November 9, 2020, and February 1, 2021. Experts were invited to take part in each round regardless of whether they had participated in previous rounds (Johnson et al., 2020). Participants received a preparation email 1 week prior to the first Delphi round, in which they were informed that the questionnaire would be sent to them as an electronic version via email unless they preferred a paper-and-pencil questionnaire. Based on the piloting, completion of the questionnaire was estimated to take approximately 30 minutes. The response deadline was set at 2 weeks. Those stakeholders who did not reply within 1 week received a follow-up email or call. This procedure was repeated for the second and third rounds.

### Procedure and Analysis

For Factors 2 to 8, responses were evaluated using Borda voting, a sum score based on given ranks. This method of analysis was chosen because it enabled a forced choice and a more accurate analysis of the participants’ preference structure than plurality voting (Poundstone, 2008). When a participant placed an option in the least relevant position, 1 point was awarded. The next relevant position was awarded 1 point more for each higher rank, resulting in the highest number of points for Rank 1 (most preferred). The sum score for an answer option was calculated by adding the points of all participants for each option within a factor. Table 1 demonstrates how Borda voting looks in practice.

**Table 1.** Example of Borda Voting in Practice.

Number of voters	Preference
	4 3 2 1 0 ←Inverse rank points
4	A > B > C > D > E
3	B > A > E > D > C
2	E > A > B > C > D
1	C > E > A > D > B

*Note*. Participants rank options according to their preferences. Each option corresponds to inverse points of 0–4 (Borda count). In the top line, A was ranked number 1, so it received its inverse point, 4. In the second line, it was ranked number 2, so it received its inverse point, 3. And so on. Points were then multiplied by the number of voters who chose them, and added together to give a sum score:

A: (4 × 4) + (3 × 3) + (3 × 2) + (2 × 1) = 32.

B: (3 × 4) + (4 × 3) + (2 × 2) + (0 × 1) = 29.

C: (2 × 4) + (0 × 3) + (1 × 2) + (4 × 1) = 14.

D: (1 × 4) + (1 × 3) + (0 × 2) + (1 × 1) = 8.

E: (0 × 4) + (2 × 3) + (4 × 2) + (3 × 1) = 17.

The option with the highest sum score wins. Option A received the most points and therefore wins.

A priori criteria for consensus were defined. For Factor 1, a particular BCT would be discarded if 67% or more of no-votes were given. For Factors 2 to 8, a clear consensus was defined if *all* stakeholders agreed to assign an answer option as Rank 1. In this case, a clear preference is found, and the factor would be excluded from the next Delphi round (Jünger et al., 2017). We defined a threshold of 67% for the most preferred options within each factor to carry them forward to the next Delphi round (van Lier et al., 2018). This is a common threshold for Delphi studies, but if 67% led to a cutoff between two answer options that were too close in points to represent a clear cut, a 75% threshold was used instead (Jünger et al., 2017).

Response items in subsequent Delphi rounds were dependent on the analysis of previous responses. Items with high agreement (>67% or >75%) were carried forward to the next Delphi round, while items that did not pass the threshold were discarded. In so doing, the number of response items was gradually reduced to achieve consensus on preferred factors. In subsequent Delphi rounds, the remaining answer options for each factor were preceded by a brief feedback report highlighting which answer options were excluded. Finally, to ensure clear preferred options for the third Delph round, the Borda counts were amended by giving the option ranked in the second position double the points of the option ranked third, and the option ranked in the first position double the points of the option ranked second (van Lier et al., 2018).

### Handling Missing or Incorrect Data

If an answer option was inadvertently selected twice, we used mean score imputation to account for it, which occasionally resulted in a decimal figure instead of a whole number. In the case of participants missing a full question or completing the questionnaire incorrectly, they were contacted and requested to correct this.

## Results

Of the 17 stakeholders invited to participate in Delphi Round 1, 1 stakeholder (a policymaker) withdrew from the study after receiving the first questionnaire, leaving a total of 16 participants who took part in the first round. Thus, a response rate of 94% (16/17) was achieved and maintained for all three Delphi rounds. Table 2 provides an overview of the composition of the expert panel.

**Table 2.** Consistent Composition of the Expert Panel Across All Three Delphi Rounds.

Stakeholder characteristics (*n* = 16)			
Stakeholder group	Clinical experts (*n* = 7)	Patients (*n* = 3)	Policymakers (*n* = 6)
Age, years (mean)	44.9	67	58.5
Sex: male (female)	4 (3)	3 (0)	4 (2)
Years of experience (mean)	12	14.3	13.2

Below, the stakeholders’ preferred choices for each of the eight factors are presented alongside the response items that were discarded from one Delphi round to the next due to low stakeholder consent. Finally, the most preferred option for each factor identified in Delphi Round 3 is presented. For Factors 2 to 8, in the vast majority of cases, the 67% threshold was applied. In those cases where the 75% threshold was applied instead, it is explicitly stated.

### Content of the Intervention (Factor 1)

None of the 11 BCTs presented received more than 67% “no” responses. All BCTs were therefore kept as potential self-care measures to be included in the final intervention manual.

### Characteristics of the Interventionist (Factor 2)

At the beginning of Delphi Round 1, 10 response items were available for selection. Stakeholders’ preferences for who should deliver the intervention resulted in “HF specialist nurses” receiving the highest number of points (119.75). Three options did not make it to Round 2 due to low stakeholder agreement: “nurses” (66 points), “nurses and carers” (62.5 points), and “physician assistants” (translation from a German-specific qualification known as “*Medizinische Fachangestelle*”; 51.75 points). In Delphi Round 2, “HF specialist nurses” still remained the top choice with 85.25 points, followed closely by “general practitioners” (GPs; 77 points). The following two options were eliminated in Round 2: “physician assistants and GPs” (57.75 points) and “trained family members” (43.75 points). In the third and final Delphi round, “HF specialist nurses” was the preferred interventionist (118 points), with “GPs” as the second preferred option (95 points).

### Characteristics of the Target Population (Factor 3)

In Delphi Round 1, 15 of the 16 stakeholders expressed their preference that “patients *and* relatives” should both receive the intervention rather than patients only. In terms of which patient group the intervention was most appropriate for, five response items were available. Stakeholders’ preference was for “individual assessment needed” (60.5 points). The following two answer options were eliminated in Round 1: “New York Heart Association (NYHA) Class I-II” (43.5 points) and “patients under 75 years” (32 points). In Delphi Round 2, “individual assessment needed” remained the top choice with 40.5 points, followed by “NYHA I-IV” (30.5 points). A further response option was eliminated in Round 2: “NYHA I-III,” which scored merely 25 points. In Delphi Round 3, “individual assessment needed” was the preferred choice (27 points), followed by “NYHA I-IV” (21 points).

### Delivery Location (Factor 4)

At the beginning of Delphi Round 1, six response items were available for selection. The preferred location for the intervention to take place was in an “outpatient HF clinic” (66 points). The following two answer options did not make it to Round 2: “rehabilitation clinic” (48 points) and “hospital” (41 points). In Delphi Round 2, “outpatient HF clinic” remained the preferred choice along with “GP practice,” which both received 43 points. The response option “cardiologist practice” was eliminated since it obtained only 35 points. In Delphi Round 3, “outpatient HF clinic” (43 points) was the preferred delivery location, with the follow-up option being “GP practice” (35 points).

### Mode of Delivery (Factor 5)

At the beginning of Delphi Round 1, five response items were available for selection. Stakeholders primarily preferred the option “general information in group session, more detailed information individually” (66 points). Two answer options did not receive enough points to move to Round 2 and were thus eliminated: “individual session” (38 points) and “group session” (25 points). In Delphi Round 2, “general information in group session, more detailed information individually” again received the highest acceptance (39 points), followed by “individual session first, then group session” (29 points). Despite applying the 75% threshold, “group sessions first, then individual sessions if needed” (28 points) did not receive enough points to move to the third round. In Delphi Round 3, “general information in group session, more detailed information individually” (27 points) was the preferred mode of delivery.

### Intensity (Factor 6)

At the beginning of Delphi Round 1, six response items were available for selection. The item “30 minutes” for the length of an intervention session was the preferred option that received the most points (81). The ensuing two options for intervention length did not make it to Round 2: “15 minutes” (43.5 points) and “2–3 hours” (31 points). In Delphi Round 2, “30 minutes” again received the most points (52). The answer option “90 minutes” (24 points) was eliminated due to low stakeholder agreement. In Delphi Round 3, “30 minutes” (52 points) was the preferred option, followed by “20 minutes” (31 points).

### Duration (Factor 7)

At the beginning of Delphi Round 1, nine response items were available for selection. The option for intervention duration that received the most points was “every 2 weeks for 1–3 months, then monthly for 6 months” (115 points). For this factor, the 75% threshold was applied again. Consequently, two answer options were eliminated: “weekly” (51 points) and “every six months” (35 points). In Delphi Round 2, the answer options with the highest number of points shifted to “twice/week in Week 1, once/week in Weeks 2–4, then every 2 weeks, then monthly, then once/quarter” (83 points). Due to low stakeholder agreement, the following two response items were eliminated: “2-day intensive training, then follow-up training after 3–4 weeks” (51 points) and “every 3 months” (42 points). In Delphi Round 3, the most preferred option was “every 2 weeks for 1–3 months, then monthly for 6 months (111 points).

### Format (Factor 8)

At the beginning of Delphi Round 1, seven response items were available for selection. The intervention format that scored the highest was “training” (translation from the German term “*Schulung*”; 101 points). Two answer options were eliminated due to low stakeholder agreement: “short brochure” (53 points) and “comprehensive handbook” (43 points). In Delphi Rounds 2 and 3, “training” again was the most preferred option, scoring 71 points and 155 points, respectively. The second preferred option in Round 3 was “telephone consultation” (83.5 points).

Table 3 provides a detailed summary of all the Delphi results from each round.

**Table 3.** Detailed Summary of the Delphi Results Round by Round for Factors 2 to 8.

Factor (No.)	Round 1	Round 2	Round 3
Interventionist (#2)		^a^	
HF specialist nurse	119.75	82.25	**118**
Family doctor	106.25	77	95
Specially trained MFA	107.75	59.5	71
Cardiologist	101	64.25	56
Nurse and doctor	89	60.5	44
MFA and family doctor	90	[57.75]	
Trained family members	86	[43.75]	
Nurse	[66]		
Nurses and carer	[62.5]		
MFA	[51.75]		
Target group (#3)		^a^	
Individual assessment needed	60.5	40.5	**27**
NYHA I-IV	51	30.5	21
NYHA I-III	53	[25]	
NYHA I-II	[43.5]		
Younger than 75 years	[32]		
Location (#4)		^a^	
Outpatient HF clinic	66	43	**43**
Family doctor practice	59	43	35
Patient’s home	62	39	34
Cardiologist practice	60	[35]	
Rehabilitation clinic	[48]		
Hospital	[41]		
Mode of delivery (#5)		^a^	
General information in a group, more detailed information individually	66	39	**27**
First individual, later group	59	29	21
First group, later individual	52	[28]	
Individual only	[38]		
Group only	[25]		
Intensity (#6)		^a^	
30 minutes	81	52	**52**
20 minutes	59.5	36	31
45–60 minutes	70	48	29
90 minutes	51	[24]	
15 minutes	[43.5]		
2–3 hours	[31]		
Duration (#7)	^b^	^a^	
Every 2 weeks for 1–3 months, then monthly for 6 months	115	79	**111**
Twice/week in Week 1, once/week in Weeks 2–4, then every 2 weeks, then monthly, then once/quarter	105	83	94
Four to six times for basic training within 2–4 weeks, then phone call every 2 months for 1 year	105.5	66	68
Twice/month	86	64	56
Once/month	90.5	63	55
2-day intensive training, then follow-up training after 3–4 weeks	67	[51]	
Every 3 months	65	[42]	
Weekly	[51]		
Every 6 months	[35]		
Format (#8)	^a^	^b^	
Training	101	71	**155**
Telephone consultation	79	47	83.5
Self-help group	61	44	64
Lecture	57.5	42	55
Digital offer	[53.5]		26.5
Short brochure	53	[36]	
Comprehensive handbook	[43]		

*Note.* The options in square brackets are the ones that were removed from the Delphi questionnaire due to low agreement. The bold underlined numbers indicate the most preferred option HF = heart failure; MFA = *Medizinische Fachangestelle* (physician assistant); NYHA = New York Heart Association functional classification.

a Both the 75% and the 67% threshold led to the same result. ^b^The 75% rule was applied.

## Discussion

The aim of the study was to define the eight factors described by Davidson et al. (2003) to successfully implement an HF self-care intervention in the German health care system. To this end, we used a ranking-type Delphi study to obtain consensus among key stakeholders.

In the final round of the Delphi study, stakeholders preferred nurses with a specialization in HF as the most suitable interventionists and an HF outpatient clinic as the most suitable location. This is in line with findings from studies of other chronic conditions. For example, Crowe et al. (2019) found that for diabetes, nurse-led interventions were efficient and cost-effective. In Germany, however, the title “HF nurse” is not regulated, and training curricula vary by their contents and comprehensiveness (Baldewijns et al., 2019). Among other things, the roles and responsibilities of HF nurses are influenced by the location in which they work (Riley et al., 2016). HF nurses could, for instance, be advanced practice nurses (APNs) who hold a university degree at the master’s or doctoral level (Schmitte, 2020). Advanced practice nursing is characterized by a high degree of professional autonomy and independent practice (Schmitte, 2020). APNs have the education and competence to autonomously deliver and coordinate patient care as a substitute for doctors. Nevertheless, such academic-practitioner nurses are not yet widely available in Germany, since the necessary adjustments to legal frameworks and structures are still absent (Schmitte, 2020). On the other hand, there are also substantial efforts to implement an area-wide network of HF units in Germany (Störk et al., 2020) that are staffed with physician assistants (*Medizinische Fachangestelle*) who undergo an additional, HF-related training ranging from 32 to 80 hours of theoretical lessons, leading to the title “specialized assistant in HF” (Ertl et al., 2016). Yet another education program for nurses comprises 120 hours of theoretical lessons and 80 hours of practical education, leading to the title “HF nurse” (Baldewijns et al., 2019). In comparison to the education received by APNs, these trainings are much shorter and based on the delegation principle—that is, specialized assistants in HF or HF nurses working under the supervision of a cardiologist or GP. Overall, different titles are used for the same functions, and use of the same title does not automatically relate to the same function or grade (Lovink et al., 2020). Despite careful scrutiny of the responses, different stakeholders had different ideas of what is meant by an HF nurse. Because of this variability in the nursing profession in Germany, the exact training prerequisite for HF nurses who deliver the intervention needs to be further elaborated.

The vast majority of stakeholders were of the opinion that patients should be included in the intervention based on an individual assessment rather than their NYHA class. Such an assessment would enable identification of patients who could potentially benefit from a self-care intervention, based on their needs and ability to participate. This could be measured with validated instruments such as the European HF Self-Care Behaviour Scale (Jaarsma et al., 2009; Vellone et al., 2014) or the revised Self-Care of HF Index (Riegel et al. 2019), combined with individual assessments of patients’ physical and psychological capability (Hammash et al. 2017; Riley et al., 2016). Furthermore, stakeholders emphasized that carers should be included in the intervention as well. Strachan et al. (2014) also highlighted the importance of including carers of patients with HF in health promotion interventions since they already assist the patients in many ways. Yet, they argue, carers may lack specific knowledge about HF, which if improved with training could make their assistance more effective. For the development of an HF self-care intervention in Germany, we will therefore include both patients and carers, and will incorporate individual assessments to determine which patients could benefit from the intervention.

The current study further revealed that the intervention should be delivered in a mixture of group and individual sessions, where general information should be provided in interactive group sessions and more individual information should be discussed individually. Benefits of group sessions have been examined: Patients with HF who participate in group trainings gain a better understanding of the disease and can rely on peer support from their fellow patients with HF (Sookhoo et al., 2013). The HF intervention will therefore be designed to enable scheduling of group sessions for general information like symptom recognition, while one-to-one appointments can be made for information specific to the individual, such as how to incorporate particular lifestyle changes into one’s current daily life. Last, stakeholders pointed to training as the main intervention format, while telephone support could serve as an adjuvant intervention format since it has been shown to be efficient for improving self-care in patients with HF and is potentially more cost-effective (Inglis et al., 2015).

Our study can be used as a practical example of how to involve key stakeholders’ opinions when designing a health care intervention. Reflecting stakeholders’ opinions in a ranking scenario provides insight into their preference structure. Capturing this preference structure with the Borda count method ensures that stakeholder opinions are more accurately reflected than a plurality vote would allow (Poundstone, 2008). It also leaves less opportunity for strategic voting—that is, prioritizing certain options to put others at a disadvantage (Poundstone, 2008).

### Strengths and Limitations

A key strength of this study is the involvement of three different stakeholder groups (Craig et al., 2018; O’Cathain et al., 2019). It may appear as if patients and carers are underrepresented since only 3 participated. However, at a previous stage of this study, 31 patients with HF were interviewed regarding the intervention; their suggestions have therefore already been incorporated (Whittal et al., 2020). Another strength is that we obtained a consistently high response rate of 94% in all three Delphi rounds. This demonstrates that the stakeholders were highly motivated to engage in the study despite the challenging circumstances caused by the COVID-19 pandemic.

Despite the strengths mentioned above, some limitations must be acknowledged. For instance, we did not involve policymakers who represent private health insurance. About 10% of the German population are covered under private health insurance, therefore we cannot draw conclusions for intervention implementation in the entire German health care system. Nevertheless, if the intervention is successfully financed in the statutory health insurance scheme, it is likely that it will be also covered in private health insurance. Furthermore, the inclusion of a delayed, but valid, questionnaire after the Round 1 analyses had been completed could be perceived as a limitation. Fortunately, this only affected Factor 8, in which “digital offer” was eliminated instead of “short brochure.” This was corrected by reintroducing “digital offer” as a response option in Round 3. Last, it is important to note that this eight-factor framework has not been thoroughly tested in practice. Future researchers could further apply and evaluate this framework.

### Implications for Practice and Future Research

Several practical implications can be drawn from this Delphi study. First, taking the local setting into account when developing complex interventions is crucial to improve the chances of implementation success (O’Cathain et al., 2019). The involvement of local key stakeholders’ experience-based insights can shed light on the practicalities of implementing an intervention in real-world practice. Research seeking to implement interventions should involve key stakeholders and understand the context for the intervention (Nilsen & Bernhardsson, 2019).

Second, this study demonstrates how a theoretically based intervention can be adapted for a local context. The basis of the intervention for the general HF population had already been developed, and the questions addressed here about tailoring it for effectiveness in the local context were a subsequent step. For research and practice, this method could be adopted: developing a base intervention and then, via interviews and a Delphi study with key stakeholders, making the necessary adaptations for local settings.

The field of implementation science, which seeks to improve the bridge between science and practice, asserts that it is important to strengthen research so that findings can be successfully applied in real-world settings (Swiss Implementation Science Network, 2021). Future research could make use of methods such as the one outlined in this article to strengthen the potential of scientific interventions to be successfully implemented in real-world practice.

## Supplemental Material

sj-docx-1-heb-10.1177_10901981211043116 – Supplemental material for Determining Contextual Factors for a Heart Failure Self-Care Intervention: A Consensus Delphi Study (ACHIEVE) Supplemental material, sj-docx-1-heb-10.1177_10901981211043116 for Determining Contextual Factors for a Heart Failure Self-Care Intervention: A Consensus Delphi Study (ACHIEVE) by Amanda Whittal, Isabell Ehringfeld, Paula Steinhoff and Oliver Rudolf Herber in Health Education & Behavior
