# Supplementary material for: Determining Contextual Factors for a Heart Failure Self-Care Intervention: A Consensus Delphi Study (ACHIEVE)
Source: Health Educ Behav. 2021 Oct 4;51(2):311–20. doi: 10.1177/10901981211043116 (PMC10981183; doi:10.1177/10901981211043116)
Supplement: sj-docx-1-heb-10.1177_10901981211043116 – Supplemental material for Determining Contextual Factors for a Heart Failure Self-Care Intervention: A Consensus Delphi Study (ACHIEVE) [file sj-docx-1-heb-10.1177_10901981211043116.docx]

**Appendix 1: ACHIEVE Study Delphi Questionnaire - Round 1**
Thank you for your participation in this consensus exercise!

The aim of this questionnaire is to produce consensus on the best possible options for key factors related to the successful implementation of an intervention for improving heart failure self-care within the German health system.

For each question below, we provide a brief overview of what we’re asking you, followed by a visual matrix. The matrix summarizes the responses we received from all 18 experts during the interviews, and includes existing scientific quantitative (meta-analysis) and qualitative (meta-synthesis) evidence in relation to this topic. When there is no existing scientific literature on the topic, we have written ‘no relevant findings'.

Against the background of this information, we ask you to think over the question once more. You are then provided with answer options derived from the interviews, and we ask that you rank them in order of preference for the German context according to your experience; 1 being the most preferred, the highest number being the least preferred. In two instances, we will ask you to provide yes/no answers instead of ranking. At the end of each question there is a textbox for you to write anything additional you wish to say in relation to the question or your answer. This information will not be analysed, but will be used as a means of communication between you and us, so that we ensure you understand clearly what we have asked, and we understand clearly your responses.

Please note that for practical purposes, only the masculine form is used throughout the questionnaire.

This should take approximately 30 minutes of your time.

You are one of our 18 experts, thus, your opinion makes a substantial difference to this study. Your participation and contributions to all three questionnaire rounds are greatly appreciated and will contribute to the success of this study.

Many thanks again.

1. **Behaviour change measures (“Content of the intervention”)**

During our interviews, we asked which behaviour change measures you found more or less important from our long list. In some instances we received ambivalent answers.

We received **ambivalent answers** in relation to the self-care measures listed in the top row of the matrix below. Besides your expert opinion, in the second row, any available quantitative scientific evidence in relation to that behaviour change measure is listed. In the third row, any available qualitative scientific evidence in relation to that answer option is included. Please read the chart carefully to get a picture of what answers were stated in the interviews, and what the scientific literature says. Then, for each of the possibilities below, indicate either ‘yes’ if you would like this answer option to be included as a potential self-care measure or ‘no’ you if would **not** like this option to be included as a potential self-care measure.

*Descriptions of non-self-explanatory behaviour change measures (indicated with a star*) are provided below the matrix.

| Your answers | Digital resources* | Teach-back method* | Involve psychologist for additional support if necessary | Self-help groups* | Differentiating symptoms from other comorbidities | Monitoring feelings* | Re-attribution* | Teaching to read food labels | Post-it reminders on fridge* | Motivational interviewing* | Cognitive behavioural therapy* |
| --- | --- | --- | --- | --- | --- | --- | --- | --- | --- | --- | --- |
| Quantitative meta- analysis findings | Systematic review conducted, inconclusive evidence | No evidence available | No evidence available | No evidence available | No evidence available | No evidence available | No evidence available | No evidence available | No evidence available | No evidence available | No evidence available |
| Qualitative meta- synthesis findings | No evidence available | No evidence available | Perception-based strategies (cognitive, emotional or psychosocial ways to cope with condition) may support self-care adherence | No evidence available | No evidence available | No evidence available | Perception-based strategies (cognitive, emotional or psychosocial ways to cope with condition) may support self-care adherence | No evidence available | No evidence available | Perception-based strategies (cognitive, emotional or psychosocial ways to cope with condition) may support self-care adherence | Perception-based strategies (cognitive, emotional or psychosocial ways to cope with condition) may support self-care adherence |

**Behaviour change measures descriptions**

*** Digital resources:** providing tools such as recommended internet sites, SMS reminders, or automated email reminders as a supportive
 addition to the main intervention format.

*** Teach-back method:** a method used to confirm whether a person understands what is being explained to them. If the person understands,
 they are able to explain or "teach-back" the information correctly.
*** Self-help groups:** groups in which patients can come together for peer support and learning as a supportive addition to the main intervention format.

*** Monitoring feelings:** patients keep a written or mental record of their feelings in situations related to their illness. E.g. how they feel emotionally if they do or do not perform a self-care behaviour.

*** Re-attribution:** replacing one way of thinking with another. E.g. suggesting a person see the positive in a situation instead of the negative.

*** Post-it reminders on fridge:** intended as a reminder for patients who perform self-care inconsistently and have difficulty prioritising it.

*** Motivational interviewing:** for patients who have difficulty deciding what to do or taking responsibility, motivational interviewing is a psychological technique that helps elicit the patients’ own views on what matters to them, what their personal barriers are, and how they can realistically take better control and care.
*** Cognitive behavioural therapy:** a psychological tool for people with anxiety or depression to help them replace and manage thoughts that
 do not serve them.

Please select your preference for each possibility below either ticking ‘yes’, you would like this answer option to be included as a potential self-care measure or ‘no’ you if would **not** like this option to be included as a potential self-care measure.

**Yes** **No** Other questions or comments about this question?

Please write them in the space below.
Digital resources……………………………………………….

Teach-back method ……………………………………………

Involve psychologist for additional support if necessary ……..

Self-help groups ……………………………………………….

Differentiation of symptoms from other comorbidities ……….
Monitoring feelings ……………………………………………

Re-attribution ………………………………………………….

Teaching to read food labels …………………………………..

Post-it reminders on fridge ……………………………………

Motivational interviewing …………………………………….

Cognitive behavioural therapy ………………………………..

| Your answers | MFAs | Specifically trained MFAs | MFAs and GPs | Nurses | HF nurses | Nurses and carers | Nurses and doctors | GPs | Cardiologist | Trained Family members |
| --- | --- | --- | --- | --- | --- | --- | --- | --- | --- | --- |
| Quantitative meta-analysis findings | No evidence available | No evidence available | No evidence available | No evidence available | No evidence available | No evidence available | No evidence available | No evidence available | No evidence available | No evidence available |
| Qualitative meta-synthesis findings | No evidence available | No evidence available | No evidence available | No evidence available | No evidence available | No evidence available | No evidence available | No evidence available | No evidence available | Carers have been found to facilitate self-care |

1. **Who should deliver the intervention? (“Characteristics of the interventionist”)**
   We asked who should be the **key person or professional group** to deliver the intervention. A **key person** is the person who holds the main responsibility for delivering the intervention. This does not exclude contributions from other professionals.

   The answer options we received from the interviews are listed in the top row of the matrix below. Besides your expert opinion, in the second row, any quantitative scientific evidence in relation to that answer option is listed. In the third row, any qualitative scientific evidence in relation to that answer option is included. Please read the chart carefully to get a picture of what answers were stated in the interviews, and what the scientific literature says.

Please rank the possibilities below from your **most preferred (1)** **to** **least preferred (10)** **key person to deliver the intervention** in the German context. To make the decision more manageable with so many options, we suggest that you **first** read all options available. Then rank your two most preferred options followed by ranking your two or three least preferred options. Finally rank the remaining options. Write your rank order number in the space provided and please ensure that all ranks are used only once.

___ Physician assistants Other questions or comments about this question? Please write them in the space below.

___ Specifically trained physician assistants

___ Physician assistants and GPs

___ Nurses

___ HF nurses

___ Nurses and carers

___ Nurses and doctors

___ General practitioners (GPs)

___ Cardiologist

___ Trained family members

1. **Who should the intervention address? (“Characteristics of the target population”)**

We asked which target group you thought this intervention should specifically address.

This question is divided into two parts. For part a), no matrix is necessary. For the matrix in part b), the answer options we received in the interviews are listed in the top row of each matrix below. In the second row, any quantitative scientific evidence in relation to that answer option is listed. In the third row, any qualitative scientific evidence in relation to that answer option is included. Please read the charts carefully to get a picture of what answers were stated in the interviews, and what the scientific literature says.

1. **Who should the intervention address?**

Tick your preferred option below with an ‘x’ regarding who the intervention should address.

Patients only

Patients and relatives

1. **What types of patients should the intervention address?**

For a better understanding, the definitions of the respective NYHA classification are listed below.

- NYHA Class 1: No symptoms and no limitation in ordinary physical activity, e.g. shortness of breath when walking, climbing stairs etc.
- NYHA Class 2: Mild symptoms (mild shortness of breath and/or angina) and slight limitation during ordinary activity.
- NYHA Class 3: Clear limitation in activity due to symptoms, even during less-than-ordinary activity, e.g. walking short distances
  (20—100 m). Comfortable only at rest.
- NYHA Class 4: Severe limitations. Experiences symptoms even while at rest. Mostly bedbound patients.

Please rank the possibilities below from your **most preferred (1)** **to** **least preferred (5)** **patients to receive the intervention** for the German context. Please ensure that all ranks are used only once.

___ NYHA 1-2 Other questions or comments about this question? Please write them in the space below.

___ NYHA 1-3

___ NYHA 1-4

___ Patients under 75 years

___ Individual assessment needed

1. **Where should the intervention take place? (“Delivery location”)**

We asked where you thought the intervention should **primarily** take place. *The primary location is the **main** location where the intervention will be delivered. This does not exclude some parts of the intervention being delivered in other locations.

Please rank the possibilities below from your **most preferred (1) to least preferred (6)** **key location of the intervention** for the German context. Please ensure that all ranks are used only once.

___ GP practice

___ Outpatient HF clinic

___ Hospital

___ Cardiologist practice
___ At home

___ Rehabilitation clinic

Other questions or comments about this question? Please write them in the space below.

1. **How should the intervention be offered? (“Mode of delivery”)**
   We asked how you thought the intervention should be offered **(group, individual, etc.)**.

The answer options we received from the interviews are listed in the top row of the matrix below. Besides your expert opinion, in the second row, any quantitative scientific evidence in relation to that answer option is listed. In the third row, any qualitative scientific evidence in relation to that answer option is included. Please read the chart carefully to get a picture of what answers were stated in the interviews, and what the scientific literature says.

| Your answers | Group | Individual first, then group | Group first, then individual if needed | General info in group, more detailed info individually | Individual |
| --- | --- | --- | --- | --- | --- |
| Quantitative meta- analysis findings | No evidence available | No evidence available | No evidence available | No evidence available | No evidence available |
| Qualitative meta- synthesis findings | Group-based self-care programmes found to enhance participation and peer support | No evidence available | No evidence available | No evidence available | No evidence available |

Please rank the possibilities below from your most **preferred (1)** **to** **least preferred (5)** **intervention delivery type** for the German context.
Please ensure that all ranks are used only once.

___ Group session

___ Individual session first, then group session

___ Group session first, then individual session if needed

___ General info in group session, more detailed info individually
___ Individual session

Other questions or comments about the above? Please write them in the space below.

1. **What is the ideal contact time for the intervention? (“Intensity”)**

   We asked what you thought the ideal **contact time** for the intervention should be.

Please rank the possibilities below from your **most preferred (1) to least preferred (6)** **intervention contact time** for the German context.
Please ensure that all ranks are used only once.

___ 2 – 3 hours Other questions or comments about this question? Please write them in the space below.

___ 90 minutes

___ 45 – 60 minutes

___ 30 minutes

___ 20 minutes
___ 15 minutes

1. **How frequently should the intervention be offered? (“Duration”)**

We asked **how frequently** you thought the intervention should be offered.

Because the answer options for this answer are very specific, we did not include a matrix with literature, but have just listed the answers we received in the interviews.

Please rank the possibilities below from your **most preferred (1) to least preferred (9) intervention frequency** for the German context. To make the decision more manageable with so many options, we suggest that you **first** read all options available. Then rank your two most preferred options followed by ranking your two or three least preferred options. Finally, rank the remaining options. Write your rank order number in the space provided and please ensure that all ranks are used only once.

___ Weekly

___ Once per month

___ Twice per month

___ Every three months

___ Every six months
___ 4-6 appointments within 2-4 weeks of basic training; then phone call every second month for 1 year

___ Every 2 weeks for 1-3 months; then monthly for 6 months

___ 2x/week in Week 1; 1x/week in Week 2-4; then every second week; then 1x/month; then 1x/quarter

___ Two-day comprehensive training, then follow-up after 3-4 weeks

Other questions or comments about this question? Please write them in the space below.

‘

1. **In what format should the intervention be offered? (“Format”)**
   We asked in which **format** you thought the intervention should **primarily** be offered. *A the primary form is the **main** method by which the intervention will be delivered. This does not exclude the use of some other intervention formats as well.

The answer options we received from the interviews are listed in the top row of the matrix below. Besides your expert opinion, in the second row, any quantitative scientific evidence in relation to that answer option is listed. In the third row, any qualitative scientific evidence in relation to that answer option is included. Please read the chart carefully to get a picture of what answers were stated in the interviews, and what the scientific literature says.

| Your answers | Digital | Written (handbook) | Written (flyer/ brochure) | Lecture | Training | Telephone consultation | Self-help group |
| --- | --- | --- | --- | --- | --- | --- | --- |
| Quantitative meta-analysis findings | No evidence | No evidence | No evidence | No evidence | No evidence | Telemonirtoring/ telephone support can improve heart failure self-care adherence | No evidence |
| Qualitative meta-synthesis findings | No evidence | No evidence | No evidence | No evidence | No evidence | No evidence | No evidence |

Please rank the possibilities below from your **most preferred (1)** **to least preferred (7)** **main intervention format** for the German context.
Please ensure that all ranks are used only once.

___ Digital Other questions or comments about this question? Please write them in the space below.

___ Written (comprehensive handbook)

___ Written (flyer/brochure)

___ Lecture
___ Training

___ Telephone consultation
___ Self-help group

**Thank you for your participation!**
